# Supplementary material for: Acceptance and compliance with micronutrient powder (MNP) among children aged 6–23 months in northern Nigeria
Source: PLOS Glob Public Health. 2022 Oct 17;2(10):e0000961. doi: 10.1371/journal.pgph.0000961 (PMC10022258; doi:10.1371/journal.pgph.0000961)
Supplement: S1 Dataset — (ZIP) [file pgph.0000961.s008.zip › S8_Dataset/In-depth Interviews.zip!/In-depth Interviews/020715_K_BK_AB_2_CG.a.pdf]

I: Describe for me the people in your immediate family living in your household

R: Long silent, three

I: I want to know about your children between the ages of six months to two years

R: We have a child of 1year, and the other one is two years

I: The one of two years is he a boy or a girl

R: She is a girl

I: Thank you very much, we have heard of food challenges for young children in this setting related to the seasons, like in this raining season what are the types food that are difficult to get

R: You know that I am a woman not a man; I will not really know food challenges.

I: For example there are seasons we have like mangoes available, which other foods do you have available now

R: We do not have mangoes in this our community now but I don't know of other places.

I: Like spinach is good for children, in the absent of spinach which other vegetables do you give your children them

R: In the absence of spinach I do give them moringa leaves and kenaf

I: The last time we came here we ask one woman what type of foods do you give to children she said tea, what is tea?

R: Tea is the mixture of milk, bounrvita, Lipton and sugar with hot water not cold water.

I: Last time you were given MNP to give to your child, could you tell me about your experiences with the product?

R: Is it the experiences in the body of the child?

I: Just give me a story how you have been using it

R: There was nothing that happened to him and there are no challenges

I: Since you started given him the product, what could say about his body?

R: With his body there is a little bit of improvement

I: Could you explain to me the improvement of your child health compared before using the MNP

R: Okay, there is a small improvement but in terms of any negative impact I have not seen any like diseases and he do take anything that is eatable

I: thank you very much, could you tell me about any difficulties encountered during the administering of MNP

R: There is nothing

I: Some women said they have difficulties in keeping the MNP, what about your experience?

R: Where I do keep my own MNP is different from theirs and after administering one to him in a day I usually closed it, keep it in a safe place.

I: You mean the children do not usually go there to take

R: I always keep it out of the reach of children, since he is the only person they said I should give

I: Since you started giving this product to him have you noticed any sign of illness, like vomiting, headache etc

R: No I have not noticed any sign of illness

I: Some women do say at times they usually forget to give their children MNP what about you

R: As for me I usually give him every day I have not missed a single day since I started giving him

I: Like those women that do forget to administer to their child, what advice could you give to them

R: What makes me to usually remember is when the child start crying of kunu it will now done on me that I have to administer the MNP with kunu when it is time for him to take kunu.

I: Like now if you are ask to give an advice to your colleague who usually forgets what you would tell her

R: I am not in the same house with her, if we are in the same house with her at the time I am administering the MNP to my child I will also tell her to do the same.

I: Thank you very much. Could you tell me how you do administer the MNP to your child?

R: I usually used a small cup with kunu mixed the MNP and give him

I: Which type of kunu do you give him

R: Is koko

I: what time do you normally give him?

R: In the morning after I have finished bathing him late in the morning at times he will drink and remained, I will keep the remaining when he is hungry again I will give him the remaining of the koko

I: The koko of MNP does he normally takes all or remained

R: He doesn’t drink all

I: What happen to the left over?

R: I do keep out of the reach of children and when he is hungry again I will take and give it to him.

I: At the time of administering the MNP to him, what is his reaction towards the koko

R: Initially he noticed it but when I continues giving him he quickly get use to it

I: When he noticed it what did he do?

R: He did not do anything

I: When you gave him at the time he noticed it was there any physical disposition

R: At first he wanted to reject it but when I gave him the second time he took it without any difficulty.

I: Now I want to know since you started giving him this product how if at all the consumption of MNP impacted the child's current diet like increased appetite

R: Is like the rate of his food intake has increased

I: Could you tell me which particular food has made him to eat more

R: It is the kunu although he has been taking normal breast milk.

I: Apart from kunu and breast milk which particular food does he take again

R: I told you earlier, he eat rice, tuwo but mostly takes breast milk and kunu

I: All these foods you mentioned has he increase the rate he takes them or reduced

R: He takes mostly kunu the more

I: In other communities we notice that the sharing of the MNP occurred, could you talk about any sharing of the MNP when you used it with another woman

R: I have no such experience because I was told is for two months per child

I: Okay in this household is there any child that has once been given

R: No no, not at all

l: Like now the two packets contains 60 sachets do you the 60 sachets will be enough for 60 days?

R: Frankly speaking I have not counted them, but I have never shared one into two for two days, I always give him based on the instruction given to me (one sachet per day). And if what you said is true then it will be enough

I: Why did you said it will be enough

R: Why I said it will be enough because I always give Isa one per day

R: Thank you very much. Now that you have used the MNP, could you tell me how you perceive it

R: I see as a supplement for the body

I: You know food or medicine also served as supplement to the body and you are aske what is this MNP to you what will you say

R: I will say it is a medicine

I: Why did you call it medicine?

R: Because when they brought they said is a medicine that cure tamowa

I: Now that you have used it, would you purchase it if it were cheaply on sale in this community?

R: If I have the mind to buy I will buy

I: Why will you buy it?

R: Because I have used it without seen any side effect

I: How much do you think you will be willing to pay for a sachet?

R: To me let it be fifty naira (N50)

I: I am very happy with this your rich information. Now tell me some ways that you believe would be effective for promoting MNP to caregivers in this community

R: As for me what is good for me may not be good for another person. The best way is to meet with the caregivers and talk to them.

I: In this community who are the most influence your opinions and acknowledge about feeding practice

R: You know I just recently married in this community, I wouldn't know the most influential people in this very issue it is the men that will know

I: Even if you just recently marry, but there some people when they said something in the community everybody will obey

R: It is the village head and the Imam and some of the health workers

I: Who influences your decision regarding feeding your baby with the MNP

R: It is the health worker of the clinic

I: Where will be a good place to disseminate heath information related to MNP

R: As for me I cannot really say anything, I think the district head and the Imam will be in a good position to answer that question

I: Why do you said is either the District head or Imam

R: The reason why I said it is the district head because he owns the village anything that has to do with the village must be made known to him

I: Who in your community do you trust the most for receiving messages about IYCF

R: It the father of the child and the district head

I: Apart from the father of the child and the district head, what form of media do you prefer for receiving MNP messages?

R: Let it be through radio and word of mouth

I: What made the implementation of tamowa to be successfull that you will like us to follow with this MNP

R: After the trial of tamowa people discover that it was good for their children and they started coming to get the tamowa

I: If you are ask to talk to another female caregiver about this MNP what will you tell her

R: I will tell her that I have used this MNP and there is no any side effect and that it is good

I: What will you tell her again in order for her to use it well

R: I will tell her to mix it with koko using one sachet and should keep it in a safe place

I: We have actually come to the end of this interview but i don't if you have any question about the MNP

R: I don't really know if the sachets in the pack are off to 30 because I have not counted them but i promise I will never missed a day

I: Do you have any suggestion for the MNP

R: My own advice is that should not to share the MNP with another child and that a sachet should not be share for two days.

I: Thank you very much for your contributions.
